# Directional Spread of Alphaherpesviruses in the Nervous System

**DOI:** 10.3390/v5020678

**Published:** 2013-02-11

**Authors:** Tal Kramer, Lynn W. Enquist

**Affiliations:** 1 Princeton University, Department of Molecular Biology, Princeton, NJ 08544, USA; E-Mail: tal.kramer@childrens.harvard.edu; 2 Children’s Hospital Boston and Harvard Medical School, F.M. Kirby Neurobiology Center, Boston, MA 02115, USA

**Keywords:** alphaherpesvirus, herpes simplex virus, pseudorabies virus, axonal transport, directional spread, cytoskeleton

## Abstract

Alphaherpesviruses are pathogens that invade the nervous systems of their mammalian hosts. Directional spread of infection in the nervous system is a key component of the viral lifecycle and is critical for the onset of alphaherpesvirus-related diseases. Many alphaherpesvirus infections originate at peripheral sites, such as epithelial tissues, and then enter neurons of the peripheral nervous system (PNS), where lifelong latency is established. Following reactivation from latency and assembly of new viral particles, the infection typically spreads back out towards the periphery. These spread events result in the characteristic lesions (cold sores) commonly associated with herpes simplex virus (HSV) and herpes zoster (shingles) associated with varicella zoster virus (VZV). Occasionally, the infection spreads transsynaptically from the PNS into higher order neurons of the central nervous system (CNS). Spread of infection into the CNS, while rarer in natural hosts, often results in severe consequences, including death. In this review, we discuss the viral and cellular mechanisms that govern directional spread of infection in the nervous system. We focus on the molecular events that mediate long distance directional transport of viral particles in neurons during entry and egress.

## 1. Introduction

### 1.1. Alphaherpesvirinae

Herpesviruses are characterized by a large double-stranded DNA genome, a complex enveloped virion and the ability to establish a latent phase as part of their lifecycle in their hosts [1]. The mammalian herpesviruses belong to the Herpesviridae family and can be classified into three subfamilies, including the alphaherpesvirinae, betaherpesvirinae and gammaherpesvirinae. Members of each subfamily are distinguished based on their genome content, the cell type where latency is established in the host and the length of their productive replication cycle [1]. Alphaherpesviruses have the broadest host range [1]. These viruses are pantropic and neuroinvasive [2,3]. Once in the peripheral or central nervous system, infection can spread within chains of synaptically connected neurons. This review addresses the molecular mechanisms underlying neuronal invasion and directional spread of alphaherpesvirus infections.

### 1.2. Model Alphaherpesviruses

The human alphaherpesviruses include herpes simplex virus types 1 and 2 (HSV-1 and HSV-2) and varicella zoster virus (VZV). Furthermore, well-studied veterinary alphaherpesviruses include bovine herpesvirus type I (BHV-1), equine herpesvirus type I (EHV-1) and pseudorabies virus (PRV) [4]. Here, we focus primarily on HSV-1 and PRV, since both viruses have served as powerful models for understanding the molecular details of viral spread and pathogenesis, both *in vitro* and *in vivo*. In particular, several features make PRV an excellent model for studying alphaherpesvirus neuronal invasion and spread. First, PRV shares a similar genome structure to VZV, HSV-1 and HSV-2, and many of its gene products are functionally homologous to those encoded by these human pathogens [5,6]. Second, PRV can be used to infect a wide variety of experimental animal models and cell types. While its natural host is the adult swine, PRV infects a remarkably broad host range of vertebrates, with the exception of higher order primates, such as humans and chimpanzees [4]. Third, PRV readily grows to high titers and is easy to manipulate in the laboratory. Purified viral DNA or bacterial artificial chromosomes carrying the entire viral genome are infectious [7,8], and sophisticated molecular biological techniques to replace and manipulate PRV genes are well established. Fourth, working with PRV poses little to no risk for laboratory workers. Fifth, PRV serves as an excellent model for understanding the pathogenic consequences of alphaherpesvirus infections in animal models. Symptoms include severe pruritus and self-mutilation (known as the “mad itch”), as well as loss of motor coordination and ataxia. Infection of non-natural hosts with wild-type PRV is uniformly lethal [4]. Finally, because PRV spreads directionally and faithfully within chains of synaptically connected neurons, it has been used extensively as a powerful tool for studying the architecture of mammalian neuronal circuits [9].

### 1.3. Neuronal Architecture, Directional Spread of Infection and Alphaherpesvirus Pathogenesis

**Figure 1:**
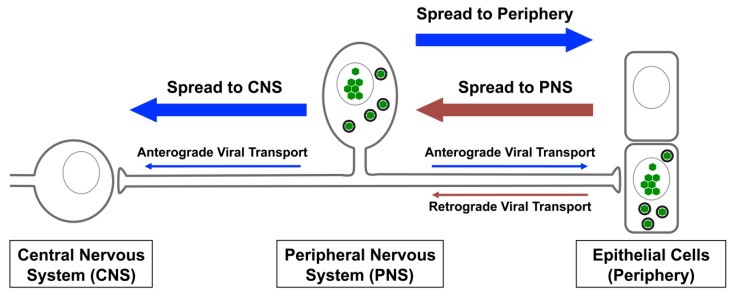
Directional spread of alphaherpesvirus infection in the mammalian nervous system. In their hosts, alphaherpesvirus infections typically initiate at peripheral sites, such as mucosal epithelia. Next, viral particles enter at the termini of sensory neurons of the peripheral nervous system (PNS). These particles are transported long distances along axons in the retrograde direction towards cell bodies, where the genomes are deposited in the nucleus to establish lifelong latency. Following reactivation from latency, new viral particles are assembled and transported towards sites of egress. Typically, infections spreads in the anterograde direction back out towards the periphery. This is essential for spread between hosts. Infection may also spread *trans*-neuronally, from the PNS to the central nervous system (CNS). Spread of alphaherpesvirus infection into the CNS is associated with lethal encephalitis.

Spread of alphaherpesvirus infection occurs between synaptically connected neurons over long distances. Prior to entering the nervous system, alphaherpesviruses typically infect and replicate in somatic cells (such as epithelia) (Figure 1). Replication in these cell types is then followed by transmission of viral infection into neurons. To invade the nervous system of their hosts, viral particles enter nerve termini of the PNS and undergo retrograde transport towards the cell body, where they initiate a lifelong latent infection [10]. Following reactivation from latency, anterograde spread of infection is dependent upon axonal sorting and transport of viral particles back out towards the periphery (Figure 1) [11]. Occasionally, the infection can spread into the CNS, an event that is often associated with the fatal consequences of alphaherpesvirus infection, such as encephalitis [12]. Thus, targeted directional transport of viral particles in neurons, while poorly understood, is an integral part of the viral lifecycle.

As a consequence of their ability to spread from the PNS to the central nervous system (CNS) or to infect peripheral tissues of non-neuronal origin, alphaherpesviruses are the causative agents of a variety of neurological diseases and disorders [12]. In humans, HSV-1 and HSV-2 infections commonly result in recurrent epidermal lesions (cold sores). Occasionally, infection may lead to more serious pathogenic consequences, such as meningoencephalitis, blindness and neonatal infections [13,14,15]. Additionally, VZV infection causes varicella (chickenpox) and herpes zoster (shingles) [16]. A variety of neurological complications are associated with VZV infections, including post-herpetic neuralgia, which is characterized by severe and constant pain in the affected dermatome [17]. Thus, the cellular and molecular underpinnings of alphaherpesvirus-inflicted neuronal damage, while poorly characterized, are central to understanding the etiology of symptoms and pathogenesis that occur as a result of infection.

Neurons are highly polarized cells with extended axonal and dendritic processes [18]. The directed trafficking of intracellular neuronal cargoes, such as ribonucleoprotein complexes (RNPs), mitochondria, synaptic vesicle precursors and other organelles, to specific subcellular domains is required to maintain both cell polarity and function [19,20]. During alphaherpesvirus infection, bidirectional transport of viral particles in axons and dendrites is essential for spread both within and between hosts [9,11]. Very little is currently known about viral particle dynamics in dendrites. The vast majority of studies have focused on sorting and transport of herpesvirus particles in axons during entry and egress [11,20,21].

*In vivo*, directional spread is likely dependent upon the architecture and connectivity of the neuron cell types that are typically infected. HSV-1, HSV-2 and VZV often establish latent infections in sensory neurons of the dorsal root and trigeminal ganglia [12]. These neurons are mostly pseudounipolar and, thus, have a bifurcated axon, such that one branch projects towards the periphery, while the other synapses with neurons of the CNS (Figure 1). Axonal sorting and anterograde transport of newly replicated particles is thought to proceed down both branches of these axons [11,22]. This presents an important conundrum. Following reactivation from latency, spread of infection towards the periphery is relatively common, whereas productive infection of the CNS is much rarer. Transsynaptic movement of viral particles likely occurs routinely from infected PNS neurons into higher order CNS neurons [23,24,25]. As evidence of this, HSV genomes are readily detectible in the CNS of infected rodents and humans [26,27,28,29,30,31]. Furthermore, Chen *et al.* demonstrated that latent HSV-1 and HSV-2 genomes could be reactivated from mouse brain stems, indicating that the CNS can be a latency site for HSV, with the potential to cause recurrent disease [23]. Similarly, latent PRV genomes have also been reported in brain tissue from adult swine, the virus’s natural host [32,33,34,35,36]. Infection of mice with a virulent strain of PRV results in peripheral disease with minimal CNS invasion, while infection with an attenuated strain invades the CNS with minimal peripheral disease [37]. Thus, invasion of the PNS always follows infection of peripheral tissues, but spread to the CNS from the PNS depends on the extent of peripheral disease. Since both HSV-1 and PRV frequently gain access to the body of their natural hosts by infecting epithelial surfaces of the oronasal region, these viruses may also invade the CNS directly via the olfactory routes [38]. In brains of patients suffering from herpes simplex encephalitis, immunohistochemical analysis showed an enrichment of viral antigen mainly in the medial and inferior temporal lobes, hippocampus, amygdaloid nuclei, olfactory cortex, insula and cingulate gyrus [39]. This localization pattern would be consistent with entry of the virus via the olfactory pathway, with spread along the base of the brain to the temporal lobes. However, another possibility is that herpes simplex encephalitis may result from viral spread from the trigeminal ganglia to the temporal and frontal cortex, which would be consistent with the known site of HSV-1 latency. Further work is needed to understand the molecular factors that determine the route of alphaherpesvirus neuroinvasion.

An important question is what are the viral or host factors that modulate or restrict the extent of viral neuroinvasion and replication in the CNS? An increasing amount of evidence suggests that the immune system plays a key role in this process by modulating reactivation from latency and viral gene expression. The innate response likely serves as the first line of defense. Toll-like receptors (TLRs) recognize pathogen-associated molecular patterns (PAMPs), such as those expressed by HSV-1. Activation of TLRs in neurons and glial cells leads to the production of cytokines that generate an antiviral response by recruiting macrophages or inducing proteins that degrade mRNA and inhibit translation. Similarly, TLR-dependent pathways are required for limiting HSV-1 replication in humans. Casrouge *et al.* previously reported that autosomal recessive mutations in UNC-93B, a transmembrane protein located within the ER, render children more susceptible to herpes simplex encephalitis by disrupting the interferon (IFN) response pathway [40]. UNC-93B functions by delivering TLR3, TLR7 and TLR9 from the ER to the endosome, the site at which they recognize PAMPs and initiate signaling cascades [41,42]. Recently, UNC-93B null patient-derived fibroblasts were differentiated into induced pluripotent stem cells (iPSCs) and then into CNS-resident cells, including neural stem cells (NSCs), neurons, astrocytes and oligodendrocytes. In this study, Lafaille *et al.* reported that UNC-93B-defective neurons and oligodendrocytes were more susceptible to HSV-1 infection compared to wild-type control cells [43]. Susceptibility was unchanged in NSCs and astrocytes. Intriguingly, similar results were obtained for cells derived from TLR3-defective neurons. These results suggest that signaling through the TLR3 pathway is necessary for efficiently modulating susceptibility to HSV-1 infection, specifically in neurons and astrocytes. This agrees with previous work indicating that TLR3-signaling is important for type I IFN responses and neuronal resistance to HSV-1 infection [44,45,46]. Collectively, these studies raise the possibility that cell types in the PNS and CNS are not equally susceptible and permissible to alphaherpesvirus infection. Furthermore, restriction of viral replication in the CNS by activation of TLRs may not be cell autonomous to CNS neurons. For example, Reinert *et al.* reported that TLR3 activation is responsible for restricting HSV-2 neuroinvasion by mediating the type I IFN response in astrocytes, thus limiting viral replication in CNS neurons [47]. The adaptive immune response also serves as an important line of defense against infections and functions to modulate viral spread in the nervous system. Viral specific T-cells are generated in response to infections within peripheral ganglia. These cells function to control viral replication and maintain latency. This topic has been recently reviewed in further detail [48].

## 2. The Alphaherpesvirus Infectious Cycle

### 2.1. Virion Attachment and Entry

**Figure 2:**
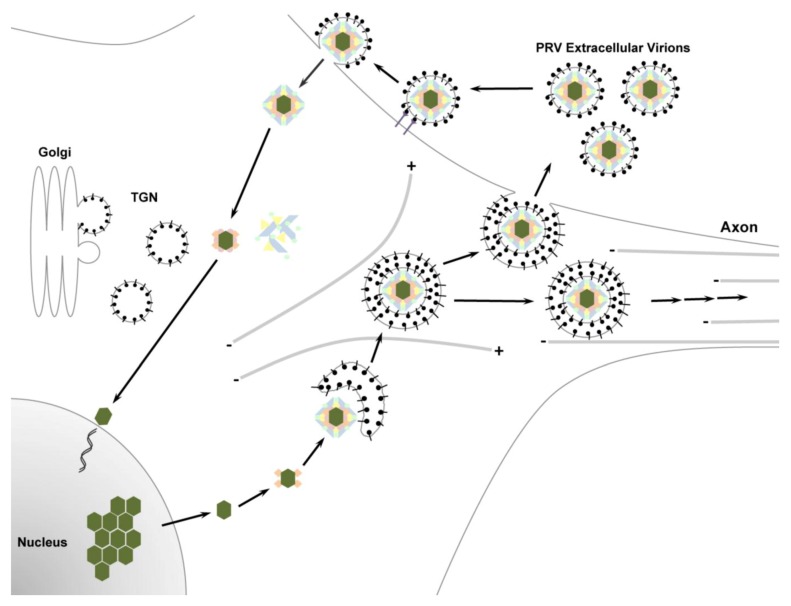
The pseudorabies virus (PRV) replication cycle. Following virion attachment and entry, viral capsids and tegument proteins are released into the cytoplasm. Many of the tegument proteins previously contained within the virion are released into the cytoplasm. Viral capsids and a specific subset of tegument proteins are trafficked towards the nucleus, where the viral genome is deposited and replicated. Capsids containing newly replicated viral genomes are released from the nucleus into the cytoplasm, where they further mature by acquiring viral and host tegument proteins. Final maturation occurs by envelopment of viral particles into vesicles derived from the trans-Golgi network (TGN) that contain viral and host membrane proteins. This process, known as secondary envelopment, results in a mature virion that is contained within a transport vesicle. Enveloped virions are trafficked towards sites of egress along the cell surface. In neurons, enveloped virions are sorted into axons and transported long distances along microtubules towards distal egress sites. At the target membrane, the transport vesicle and plasma membrane fuse, releasing a mature, enveloped PRV virion from the cell.

The directionality of spread is dependent upon the nature of the alphaherpesvirus infectious cycle (Figure 2). Infection is initiated by mature virions, which have a complex multilayered structure that is conserved among all herpesviruses [1]. At the core of the virion is a linear double-stranded DNA genome that is packaged within a proteinaceous capsid. The capsid is surrounded by a layer of viral and cellular proteins known as the tegument, which is enclosed within a phospholipid bilayer studded with viral transmembrane proteins and is known as the virion envelope [4,49]. Infection is initiated by attachment of virions to heparan sulfate proteoglycans on the cell surface through the glycoproteins gC [50,51] and gB [52,53]. The next step of HSV-1 virion entry is dependent upon glycoprotein gD, which binds receptors on the surface of the plasma membrane, including Nectin-1, herpesvirus entry mediator (HVEM) and 3-O-sulfated heparan sulfate [54,55,56,57]. For PRV, gD is required for penetration of virions into cells, but not for cell-to-cell spread and neuroinvasion [58,59,60]. Although PRV gD binds Nectin-1 [61,62], neither Nectin-1 nor HVEM are required for PRV entry and spread in fibroblasts [60]. Thus, it is likely that there are other cellular receptors for PRV. VZV does not encode a gD homolog and likely depends on different viral proteins for interacting with entry receptors [63]. VZV gE may have gD-like functions during initiation of infection through binding the insulin-degrading enzyme (IDE), a proposed VZV receptor [64,65,66]. Following virion binding, glycoproteins gB, gH and gL, mediate fusion of the virion envelope with the plasma membrane [67,68,69,70]. Penetration of viral particles into the cell triggers the release of many of the tegument proteins into the cytoplasm [71,72]. However, a specific subset of “inner tegument” proteins (UL14, UL16, UL21, UL36, UL37, Us3 and ICP0) are thought to remain associated with capsids following entry [11,73]. These capsid tegument complexes are transported towards the nucleus [71,72]. Three of these proteins (UL36, UL37 and Us3) have been shown to co-transport with incoming PRV capsids by live cell imaging and electron microscopy [10,71,72,74,75]. Recent studies have focused on the role of the large-inner tegument protein, UL36, during viral entry. UL36 serves multiple functions during infection, including transport of capsids towards the nucleus [73,76], docking of capsids to the nuclear pore [74] and release of viral genomes into the nucleus [77]. Upon entering the nucleus, the linear viral genome becomes circularized. During productive replication, circular genomes serve as the template for DNA synthesis. The initial theta replication mechanism switches to a rolling-circle mechanism in order to produce long linear concatemeric genomes that serve as substrates for genome encapsidation [1,4]. Entry of the viral genome into the nucleus also results in the initiation of a highly temporally ordered viral gene transcription cascade that is characteristic of herpesvirus infection. Viral genes can be subdivided into three classes of successively expressed transcripts (immediate early, early and late genes) [1,4]. For PRV, gene transcription is initiated by expression of the immediate early gene IE180 (the homologue of HSV ICP4), a potent transcriptional activator that transinduces the expression of viral early genes [4,78]. Early genes primarily encode enzymes that are involved in nucleotide metabolism, while late genes encode structural components that are essential for genome packaging and assembly of new virions.

### 2.2. Genome Replication and Nuclear Egress

Newly replicated viral genomes are packaged into capsids in the nucleus. This process involves cleavage of the replicated concatemeric DNA into monomeric linear genomes and subsequent packaging of genomes into capsids [79,80]. After packaging, PRV nucleocapsids are released from the nucleus by crossing the nuclear envelope and then entering the cytoplasm. The molecular details of this process have been subject to debate over recent years [81]. The widely accepted model suggests that nuclear egress is initiated when nucleocapsids bud through the inner nuclear membrane into the perinuclear space through a process known as primary envelopment. This is followed by de-envelopment (fusion) at the outer nuclear membrane and release of the nucleocapsid into the cytoplasm [82,83]. Previously, this mode of nuclear egress was thought to be unique for herpesviruses. However, Speese *et al.* recently demonstrated that nuclear egress of large ribonucleoprotein particles (RNPs) occurs though a similar mechanism, suggesting that herpesvirus nuclear egress progresses by taking advantage of an existing cellular mechanism [84]. Nonetheless, efficient nuclear egress of herpesvirus nucleocapsids is dependent upon the viral proteins UL31, UL34 and Us3 [85,86,87,88,89,90]. Recent work has shown that additional viral tegument and membrane glycoproteins are associated with primary enveloped virions [91,92,93,94]; the putative roles of these proteins during nuclear egress remain poorly understood.

### 2.3. Virion Assembly, Secondary Envelopment and Cellular Egress

Following nuclear egress, virions undergo assembly and maturation in the cytoplasm, where they acquire tegument proteins and bud into vesicles that give rise to the final envelope [49]. Membrane acquisition of tegument-coated capsids occurs through a process known as secondary envelopment. This process produces enveloped virions that are contained within a transport vesicle. The cellular membranes used for secondary envelopment of PRV and HSV-1 have been shown to contain markers for the trans-Golgi network (TGN) [95,96,97,98]. However, it is also important to note that productive herpesvirus infection results in a dramatic rearrangement of membranous organelles, including the Golgi apparatus, TGN and endosomes, such that their subcellular localizations and boundaries become less well defined compared to uninfected cells [97,99]. Thus, while the exact cellular origin of membranes used for secondary envelopment remains unknown, these membranes contain a subset of viral and cellular proteins that facilitate virion assembly and egress.

For viral particles that are targeted for egress in axons, a lively debate has arisen regarding the assembly state of these particles prior to axonal sorting [11,100,101]. The “married model” holds that virion assembly and secondary envelopment occur in the cell body prior to axonal sorting and transport towards sites of egress at distal sites [83,102,103,104,105,106,107,108,109,110,111,112,113,114,115,116,117,118]. In contrast, under the “separate model” naked capsids (nucleocapsids that associate with a subset of tegument proteins, but not membranes) and secondary envelopment membranes undergo axonal sorting and transport separately. Final assembly of these components occurs at or near sites of egress [103,119,120,121,122,123,124,125,126,127]. Kratchmarov *et al.* recently presented a comprehensive review of the published evidence that supports each of these models [100]. An important conclusion is that overwhelming amounts of data support the married model during PRV infection. Further work is necessary to assess whether axonal sorting and transport of HSV-1 follows the married or separate models or, potentially, a combination of both [103].

Virion egress occurs by fusion between the transport vesicle membrane and the plasma membrane to releases virions into the extracellular environment. The molecular mechanism underlying these fusion events is unknown, but is likely dependent upon soluble N-ethylmaleimide attachment protein receptor (SNARE) proteins that mediate exocytosis of cellular vesicles [128,129,130]. Previous studies have demonstrated that HSV-1 and PRV particles are associated and co-transport with proteins that are involved in axonal secretory and exocytic pathways [105,120]. Furthermore, we recently found that the vesicle and target SNAREs (v-SNAREs and t-SNAREs), VAMP2, SNAP-25, VTI1B and Syntaxin-6 are incorporated with membrane microdomains that contain the PRV membrane protein Us9 [131]. As discussed below, since PRV Us9 is required for anterograde spread of infection, these cellular fusion proteins represent excellent candidates for mediating targeted virion egress in axons and dendrites. 

## 3. Utilization of the Host Cytoskeleton for Directional Spread of Infection in Neurons

### 3.1. Viral Particle Movement Requires Active Transport

Many viruses, including PRV and HSV, rely on interactions with the host cytoskeleton for effective spread and transmission. Diffusion is an inefficient means for long-distance directional movement of viral particles within the cytoplasm. An HSV capsid would take over 200 years to move 1 cm by diffusion alone [132]. This timescale is far too extended for efficient movement of viral particles in mammalian cells, let alone highly elongated cells, such as neurons that extend neurites millimeters or even meters away from their cell bodies. Thus, active transport via established cellular mechanisms is required for efficient viral infection and spread.

### 3.2. The Actin and Microtubule Cytoskeletons

Both the actin and microtubule cytoskeleton have well-established roles during herpesvirus infection [21,133,134]. Actin filaments (F-actin) play an essential role in short-distance cargo movement [135,136,137,138]. Filaments are composed of G-actin monomer subunits that form two protofilaments that wrap around each other [139]. This arrangement confers an intrinsic polarity consisting of “plus” (or “barbed”) and “minus” (or “pointed”) ends. Actin filaments range between 5 to 9 nm in diameter and often form three-dimensional networks within the cell, but are concentrated at the cell cortex [140]. Directed transport of cellular cargoes is mediated by the myosin superfamily of motor proteins [141]. Several members of this large superfamily of motors have been shown to be involved in targeting proteins to axons [142,143]. 

In contrast to the actin cytoskeleton, microtubules are required for facilitating long distance transport of cargo. Microtubules are stiff cylinders composed of alpha-tubulin and beta-tubulin heterodimeric subunits that assemble through head-to-tail association to form protofilaments [144]. Typically, 13 of these protofilaments join to form a hollow microtubule that has an outer diameter of 25 nm. Because of their composition, microtubule filaments possess an inherent structural polarity. Microtubules have a highly dynamic “plus” end that is often oriented towards the cell periphery, as well as a relative stable “minus” end that is oriented towards the microtubule organizing center (MTOC) [145,146,147]. In neurons, the arrangement of microtubules is highly specialized. Axons contain microtubules that are oriented almost exclusively with plus-ends facing the axon terminus, while dendrites contain microtubules of mixed polarity [143,148]. This mode of organization is required for maintaining the identities of these distinct neuronal compartments. Two classes of molecular motor proteins mediate microtubule-dependent directional transport of cargoes. Dynein motors facilitate retrograde transport (towards the microtubule minus ends), while kinesin family motors facilitate anterograde transport (towards the microtubule plus ends) [149]. The kinesin family of motors is substantially more diverse; mammalian genomes can encode up to 45 different kinesin motors that are classified into 14 different subfamilies [143,150]. 

The actin and microtubule cytoskeletons often function cooperatively to maintain and facilitate proper cellular polarity and dynamics [151,152]. For example, the proximal axon is enriched in filamentous actin [153], as well as microtubule ends that are decorated with the microtubule end-binding (EB) proteins, EB1 and EB3 [154,155]. These structural components are anchored by the large scaffolding protein, ankyrin G, a key organizer of the axon initial segment [156]. Combined, these structural properties of the axon initial segment allow it to function as a selective filter for regulating transport of cellular components into the axonal compartment [153].

### 3.3. Retrograde Transport During Viral Entry

To gain access to the cytoplasm, incoming viral particles must get past a layer of cortical actin [157]. While virion binding to cell surface receptors triggers rearrangement of the actin cytoskeleton [158,159], the importance of this event is currently unknown. Pretreatment of cells with the actin depolymerizing agent, cytochalasin D, does not affect the dynamics of viral entry [157]. In contrast, pretreatment of cells with the microtubule depolymerizing agents, nocodazole or colchicine, results in disruption of retrograde capsid transport towards the nucleus, demonstrating that the microtubule cytoskeleton is required for entry [22,157,160]. Live cell imaging of fluorescently labeled PRV and HSV-1 capsids in cultured neurons has shown that retrograde transport is a robust process that occurs at a rate of approximately 1 μm/s [22,105,109,160,161,162]. This rate is consistent with fast axonal transport with the retrograde motor dynein [163]. Additionally, retrograde transport of capsids is salutatory and bidirectional, suggesting that kinesin motors may also be involved [105,162]. Both kinesin-1 and kinesin-2 have been shown to co-purify with partially tegumented capsids that were generated from extracellular virions and incubated in cytosolic extracts [73].

A small subset of viral capsid and tegument proteins have been implicated in recruiting motors that facilitate retrograde transport, including UL36, UL37 and VP26 (UL35) [73,74,76,164,165,166,167]. UL36 has emerged as the most likely candidate for direct motor recruitment to incoming virions. Expression of mutant UL36 proteins or injection of anti-UL36 antibodies results in a dramatic disruption of capsid delivery to the nucleus and, consequently, initiation of infection [74,76,166,168,169]. In contrast, deletion or mutation of UL37 or VP26 does not appear to have the same effect [165,170,171,172].

### 3.4. Anterograde Viral Transport and Axonal Sorting

In addition to its role during entry, UL36 is required for transport to the site of secondary envelopment and egress [173,174]. In the absence of UL36, PRV capsids are not transported along microtubules and do not undergo secondary envelopment. PRV deleted for UL37 is defective for secondary envelopment and displays reduced capsid transport (UL36 still binds capsids in UL37 null PRV) [76]. These observations suggest that UL36 and UL37 are key components of a motor protein recruitment complex that transports tegumented (or partially tegumented) capsids towards assembly sites. Presumably, these transport events depend primarily upon interactions with kinesin motors (as opposed to dynein). However, since the tegument compositions of newly produced nucleocapsids and incoming capsids appear to be similar (capsid proteins bound to UL36, UL37 and Us3), this leads to an apparent problem. How are capsids differentially targeted to the nucleus for entry or to sites of cytoplasmic envelopment for egress? Since UL36 has been shown to interact with motor proteins of opposite polarity (G.A. Smith, personal communication), it likely has the capacity to transport viral particles in both the anterograde and retrograde directions. Posttranslational modifications or binding of accessory viral tegument proteins are predicted to act as the “switch” between dynein and kinesin driven transport [175,176,177,178,179,180].

Following secondary envelopment in the cytoplasm, viral particles are further transported towards sites of egress along the cell periphery. This process is also sensitive to microtubule depolymerizing agents and, therefore, requires kinesin motors [125]. The specific viral and cellular proteins that mediate this process have not been previously identified.

In non-neuronal cells, UL36 and Us11 have been proposed as candidate viral proteins for recruitment of kinesin motors during anterograde transport of enveloped viral particles [167,181,182,183,184]. Both proteins have been shown biochemically to interact with kinesin motor subunits. However, since UL36 and Us11 are tegument proteins, they are unlikely to partake in protein-protein interactions on the cytoplasmic surface of enveloped particles. One possibility is that a subset of tegument proteins, including UL36, may be membrane-associated and available on the outer surface of enveloped particles [185,186,187,188]. A second model holds that virions are partially enveloped, thus rendering the tegument exposed to cytosolic proteins [167]. Moreover, the potential involvement of Us11 in anterograde transport of enveloped virions poses addition caveats. First, Us11 is non-essential for viral replication. Second, Us11 is not conserved in all alphaherpesviruses, including PRV [182,183]. Thus, further work is needed to identify the viral and host proteins that facilitate anterograde transport of enveloped virions.

**Figure 3:**
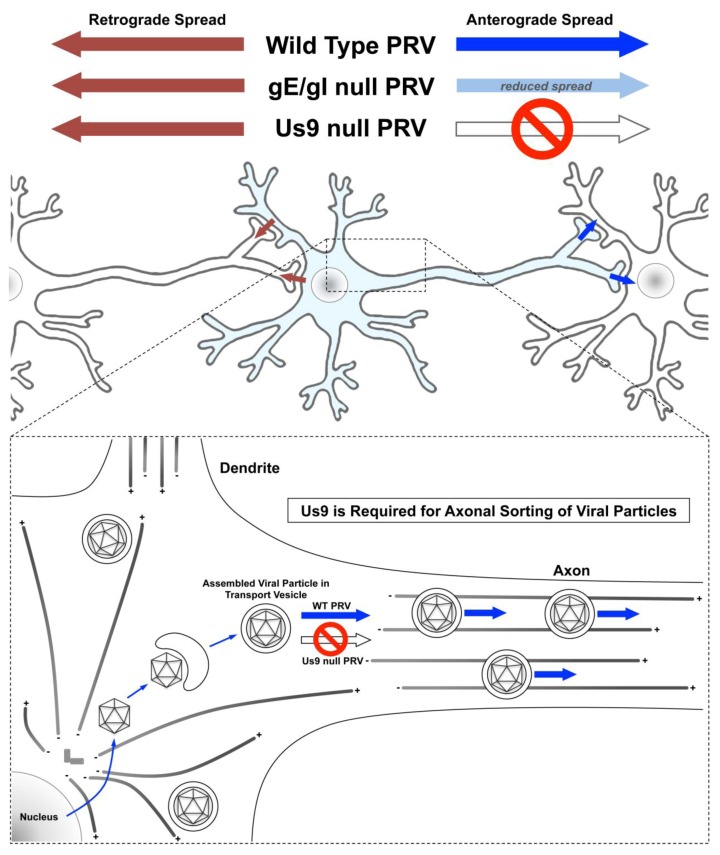
PRV Us9 and gE/gI mediate anterograde spread of infection in neurons. PRV Us9 is essential for anterograde trans-neuronal spread *in vivo* and *in vitro*. In the absence of Us9, viral particles are assembled in the cell body, but are not sorted into axons. This neuron-specific phenotype suggests that Us9 functions by recruiting a molecular motor protein, either directly or indirectly, that facilitates axonal sorting of viral particles into axons. In contrast, gE/gI null mutants are defective for anterograde trans-neuronal spread *in vivo*, but are able to spread with reduced capacity *in vitro*. gE/gI null mutants display a small plaque phenotype in non-neuronal cell types.

During neuronal infection, anterograde spread is dependent upon axonal sorting and long distance transport of viral particles. Anterograde-directed movement of viral particles is necessary for re-infection of innervated tissues following reactivation from latency and, consequently, successful spread between hosts. Three viral membrane proteins (gE, gI and Us9) are required for anterograde directed spread through neural circuits *in vivo* [37,189,190,191,192,193,194,195,196]. *In vitro*, the phenotype of individual gE, gI or Us9 null mutants are more varied (Figure 3). PRV gE and gI null mutants are able to undergo anterograde spread, but with a reduced capacity compared to wild-type [197]. Us9 null mutants display the most severe phenotype and are completely defective for anterograde transneuronal spread [108].

All three of these proteins are required for efficient targeting of virion structural components into axons [108,111,124]. gE/gI are both type I membrane proteins that form a heterodimer within the endoplasmic reticulum (ER) [195]. After leaving the ER, gE/gI are localized primarily to the Golgi apparatus, cytoplasmic vesicles and the plasma membrane [198,199]. By biotin labeling of cell surface proteins or using specific antibodies, our lab demonstrated that gE/gI undergo constitutive endocytosis from the plasma membrane and accumulate in large cytoplasmic vesicles. In porcine kidney epithelial cells (PK15), endocytosis of the gE/gI complex occurs early after infection, but stops completely after 6 hours post infection (HPI) [198]. Endocytosis of gE/gI is dependent upon the 123 amino acid cytoplasmic tail domain of gE (the full length protein contains 577 amino acids), especially a membrane proximal tyrosine-based internalization motif (at amino acids 478–481) [200,201]. Both HSV-1 and VZV gE also contain similar tyrosine-based motifs within their cytoplasmic domains that are important for endocytosis [200]. For PRV, this domain is not required for transneuronal spread of infection* in vivo* [199,201]. However, deletion of gE’s cytoplasmic domain or the full length protein results in a small plaque phenotype in non-neuronal cell types, suggesting that it is required for cell-to-cell spread in a variety of cell types [199,201]. In contrast, Us9 null mutants form plaques that are comparable in size to those formed by wild-type PRV [190]. Overall, these results imply that gE/gI and Us9 have distinct functions for mediating anterograde transneuronal spread.

Us9 is a small (106 amino acid) type II tail-anchored membrane protein that is expressed with late kinetics and is enriched within detergent resistant membrane microdomains, known as lipid rafts [202,203]. In the absence of Us9, viral particles are assembled in the cell body, but are unable to sort into axons (Figure 3) [102,108]. Moreover, the Us9 null phenotype is neuron-specific. A long held hypothesis had been that Us9 functions by interacting directly or indirectly with a kinesin motor or cellular adaptor complex that facilitates axonal sorting and transport [124,204]. Despite numerous attempts to identify viral and host proteins that interact with Us9, the molecular mechanism underlying its function remained elusive for many years [108,124,203]. We recently reported that Us9 interacts with KIF1A, a member of the kinesin-3 family of microtubule-dependent molecular motors. Our results imply that KIF1A is also required for axonal sorting and long-distance transport of viral particles towards egress sites in axons. Multiple known properties of KIF1A function are consistent with its role in mediating axonal sorting and transport of viral particles. First, KIF1A is highly expressed in neuronal tissues and is enriched in axons [205,206], thus making it an excellent candidate for facilitating anterograde spread of neurotropic alphaherpesviruses. Second, KIF1A has a well-established functional roll in axonal sorting and transport of cargoes, such as synaptic vesicle precursors and dense core vesicles [207]. Several laboratories have reported that loss of KIF1A leads to a reduced number of synaptic vesicle precursors in axons and accumulation of these organelles in cell bodies [208,209,210]. The Us9 null phenotype is strikingly similar [108,203]. Finally, KIF1A is a relatively fast kinesin motor, with average velocities of 1.2-2.5 μm/s, depending on the model system [211]. The measured average velocity of anterograde-directed PRV capsids in axons is within this range (1.97 μm/s) [212]. Similarly, the velocity of anterograde transport of VZV particles has been estimated at 1.5 μm/s [213]. However, we cannot exclude the possibility that other kinesin motors are involved in axonal transport of viral particles after axonal sorting. If so, recruitment of additional motor proteins is likely independent of Us9. 

Our results also imply that at least one additional kinesin motor is required for transport of enveloped viral particles in neuron cell bodies and non-neuronal cells. The evidence for this is as follows: (1) Retrograde transneuronal spread is unaltered during infection with PRV strains that are Us9 null [190]. (2) The Us9 null phenotype is neuron specific. Cell types that do not express KIF1A, such as epithelial cells, are susceptible and permissive to PRV infection and are capable of facilitating cell-to-cell spread of infection [124]. Both of these observations necessitate active transport of viral components towards sites of egress at the plasma membrane of cell bodies. (3) Previous studies have implicated other motor proteins in intracellular transport of viral structural components in non-neuronal cells [73,101,167,214]. Unlike KIF1A, which is expressed exclusively in neurons, these motors are expressed in a variety of cell types [143,215]. While the viral and host proteins that mediate these transport events are still unknown, kinesin-1 represents an excellent candidate, both because of its well-characterized function in transporting a broad range of cellular cargoes and its ubiquitous expression in virtually all mammalian cell types [215]. 

Viral membrane-associated proteins represent the most likely candidates for recruiting motor protein subunits to enveloped virions. In this case, deletion of these viral membrane proteins would produce a phenotype in which virions are assembled, but remain stationary and accumulate within cell bodies of non-neuronal cells (following transfection of mutant viral DNA). However, it has also been proposed that non-membrane associated viral proteins, such as UL36, may also act as receptors for kinesin motors following secondary envelopment [11,167]. This model is dependent upon UL36’s putative localization on the cytoplasmic surface of the transport vesicle surrounding intracellular enveloped viral particles, in addition to its well-characterized localization within the tegument layer of virions. Finally, it is also possible that cellular proteins mediate recruitment of kinesin-1 or other motors (besides KIF1A) onto enveloped viral particles.

### 3.5. Reorganization of the Cytoskeleton and Alteration of Organelle Dynamics During Infection

Alphaherpesvirus infections induce alterations of actin filaments, microtubules and other cellular organelles in a variety of cell types [21,133,134]. For example, the viral kinase, Us3, promotes disassembly of actin stress fibers and outgrowth of cellular protrusions that are proposed to enhance cell-to-cell spread and viral egress [216]. Consistent with this, Roberts and Baines demonstrated that the actin-dependent motor myosin Va has a role in targeting viral membrane glycoproteins to the plasma membrane [214]. Alphaherpesvirus proteins that affect the microtubule cytoskeleton include HSV-1 ICP0, a ring finger E3 ubiquitin ligase that plays a key role in establishing infection. ICP0 is multifunction and has been shown to bundle and dismantle microtubules in transfected or HSV-1 infected Vero cells [217]. 

In neurons, altered or aberrant neuronal firing activity leads to reorganization of key cytoskeletal components and changes in organelle dynamics. Two prominent examples are modification of the size and localization of the axon initial segment, as well as alteration of mitochondrial dynamics in response to increased action potential firing [218,219,220,221,222]. These changes have been implicated in a variety of neurological diseases and disorders [223,224]. Our lab previously reported that action potential firing rates dramatically increase beginning at 8-10 hours post PRV infection in cultured rat superior cervical ganglion (SCG) neurons [225]. Consequently, this results in elevated intracellular Ca^2+^ and alters mitochondrial dynamics through a mechanism involving the Ca^2+^-sensitive protein, Miro, a cellular protein that regulates mitochondrial motor activity [218,220,226]. Our findings suggest that disruption of mitochondrial motility is important for efficient spread of infection. We hypothesized that this could be due to removal of kinesin-1 from mitochondria for efficient transport of viral particles and, consequently, spread within and between hosts [226]. This hypothesis was based on two observations: (1) kinesin-1 recruitment to mitochondria through Miro is disrupted during infection and (2) PRV overexpressing Ca^2+^-insensitive Miro proteins (PRV Miro1ΔEF) is deficient for spread in both PK15 cells and SCG neurons. However, our findings do not exclude the possibility that disruption of mitochondrial motility may be important for other aspects of viral infection and spread (besides viral particle transport). One possibility is that disruption of mitochondrial dynamics through Miro’s Ca^2+^-sensitive function may be important for maintaining cell viability by blocking apoptosis. In neurons, elevated intracellular Ca^2+^ results in excitotoxic stress that leads to cell death [227]. It is remarkable that neurons cultured* in vitro* are able to survive for several days post infection [225] and do not die of excitotoxic Ca^2+^ overload. Alternatively, disruption mitochondrial dynamics during infection may interfere with cellular antiviral signaling cascades that are mediated by mitochondrial membrane proteins. For example, activation of retinoic acid-inducible gene I-like receptors (RLRs) promotes elongation of the mitochondrial network. During infection, this is important for signal transduction through the mitochondrial antiviral signal (MAVS) protein, which triggers the production of type I interferons and proinflammatory cytokines. Castanier* et al.* previously demonstrated that a human cytomegalovirus (HMCV) antiapoptotic protein that promotes mitochondrial fragmentation inhibits signaling downstream from MAVS [228]. This may be a novel HCMV immune modulation strategy.

Thus, disruption of cytoskeletal and organelle dynamics during infection may be necessary for mediating efficient viral spread. Further work is needed to identify additional intracellular changes that occur during infection and determine their potential role in promoting viral replication, growth and spread.

## 4. Concluding Remarks

Directional movement of alphaherpesvirus infection in the nervous system is essential for replication and spread within and between hosts. Efficient spread of infection in and out of the nervous system is dependent upon long distance transport of viral particles in axons and dendrites. Transport of viral particles, like other cargoes, requires the microtubule-dependent molecular motors. Retrograde transport is dependent on interactions between incoming capsids and tegument proteins with dynein motors. For anterograde spread of infection, plus-end directed kinesin motors facilitate movement of viral particles towards distal sites of egress. The protein composition of viral particles determines which motors are recruited and activated, thus determining the destination of these particles within infected cells. Additional work is needed to understand how viral complexes utilize host proteins for efficient long distance transport within their hosts. In addition, future studies will explore the significance of alterations to cellular organization, dynamics and homeostasis that occur as a consequence of infection. These types of studies are necessary for understanding the nature of alphaherpesvirus spread and pathogenesis in the nervous system.

## References

[1] Pellett P.E., Roizman B., Knipe D.M., Howley P.M., Griffin D.E., Lamb R.A., Martin M.A., Roizman B., Straus S.E. (2007). The Family: Herpesviridae a Brief Introduction. Fields Virology.

[2] Goodpasture E.W., Teague O. (1923). Transmission of the virus of herpes febrilis along nerves in experimentally infected rabbits. J. Med. Res..

[3] Goodpasture E.W., Teague O. (1923). Experimental production of herpetic lesions in organs and tissues of the rabbit. J. Med. Res..

[4] Pomeranz L.E., Reynolds A.E., Hengartner C.J. (2005). Molecular biology of pseudorabies virus: Impact on neurovirology and veterinary medicine. Microbiol. Mol. Biol. Rev..

[5] Szpara M.L., Tafuri Y.R., Parsons L., Shamim S.R., Verstrepen K.J., Legendre M., Enquist L.W. (2011). A wide extent of inter-strain diversity in virulent and vaccine strains of alphaherpesviruses. PLoS Patho..

[6] Szpara M.L., Parsons L., Enquist L.W. (2010). Sequence variability in clinical and laboratory isolates of herpes simplex virus 1 reveals new mutations. J. Virol..

[7] Smith G., Enquist L.W. (2000). A self-recombining bacterial artificial chromosome and its application for analysis of herpesvirus pathogenesis. Proc. Natl. Acad. Sci. USA.

[8] Szpara M.L., Tafuri Y.R., Enquist L.W. (2011). Preparation of viral DNA from nucleocapsids. J. Vis. Exp..

[9] Ekstrand M.I., Enquist L.W., Pomeranz L.E. (2008). The alpha-herpesviruses: Molecular pathfinders in nervous system circuits. Trends Mol. Med..

[10] Antinone S., Smith G. (2009). Retrograde axon transport of herpes simplex virus and pseudorabies virus: A live-cell comparative analysis. J. Virol..

[11] Smith G. (2012). Herpesvirus transport to the nervous system and back again. Annu. Rev. Microbiol..

[12] Steiner I., Kennedy P.G., Pachner A.R. (2007). The neurotropic herpes viruses: Herpes simplex and varicella-zoster. Lancet Neurol..

[13] Kimberlin D.W. (2005). Herpes simplex virus infections in neonates and early childhood. Semin. Pediatr. Infect. Dis.

[14] Whitcher J.P., Srinivasan M., Upadhyay M.P. (2001). Corneal blindness: A global perspective. Bull. World Health Organ..

[15] Wald A., Zeh J., Selke S., Warren T., Ryncarz A.J., Ashley R., Krieger J.N., Corey L. (2000). Reactivation of genital herpes simplex virus type 2 infection in asymptomatic seropositive persons. N Engl. J. Med..

[16] Heininger U., Seward J.F. (2006). Varicella. Lancet.

[17] Kost R.G., Straus S.E. (1996). Postherpetic neuralgia—pathogenesis, treatment, and prevention. N. Engl. J. Med..

[18] Rasband M.N. (2010). The axon initial segment and the maintenance of neuronal polarity. Nat. Rev. Neurosci..

[19] Moughamian A.J., Holzbaur E.L.F. (2012). Synaptic vesicle distribution by conveyor belt. Cell.

[20] Enquist L.W. (2012). Five questions about viral trafficking in neurons. PLoS Pathog..

[21] Zaichick S.V., Bohannon K.P., Smith G.A. (2011). Alphaherpesviruses and the cytoskeleton in neuronal infections. Viruses.

[22] Topp K.S., Meade L.B., LaVail J.H. (1994). Microtubule polarity in the peripheral processes of trigeminal ganglion cells: Relevance for the retrograde transport of herpes simplex virus. J. Neurosci..

[23] Chen S.-H., Yao H.-W., Huang W.-Y., Hsu K.-S., Lei H.-Y., Shiau A.-L., Chen S.-H. (2006). Efficient reactivation of latent herpes simplex virus from mouse central nervous system tissues. J. Virol..

[24] Tyler K.L. (2004). Herpes simplex virus infections of the central nervous system: Encephalitis and meningitis, including mollaret's. Herpes.

[25] Smith C., Lachmann R.H., Efstathiou S. (2000). Expression from the herpes simplex virus type 1 latency-associated promoter in the murine central nervous system. J. Gen. Virol..

[26] Cabrera C.V., Wohlenberg C., Openshaw H., Rey-Mendez M., Puga A., Notkins A.L. (1980). Herpes simplex virus DNA sequences in the cns of latently infected mice. Nature News.

[27] Fraser N.W., Lawrence W.C., Wroblewska Z., Gilden D.H., Koprowski H. (1981). Herpes simplex type 1 DNA in human brain tissue. PNAS.

[28] Marsden H. (1980). Herpes simplex virus in latent infection. Nature News.

[29] Rock D.L., Fraser N.W. (1983). Detection of hsv-1 genome in central nervous system of latently infected mice. Nature News.

[30] Sekizawa T., Openshaw H. (1984). Encephalitis resulting from reactivation of latent herpes simplex virus in mice. J. Virol..

[31] Sequiera L.W., Jennings L.C., Carrasco L.H., Lord M.A., Curry A., Sutton R.N. (1979). Detection of herpes-simplex viral genome in brain tissue. Lancet.

[32] Sabó A., Rajcáni J. (1976). Latent pseudorabies virus infection in pigs. Acta. virologica.

[33] Beran G.W., Davies E.B., Arambulo P.V., Will L.A., Hill H.T., Rock D.L. (1980). Persistence of pseudorabies virus in infected swine. J. Am. Vet. Med. Assoc..

[34] Wheeler J.G., Osorio F.A. (1991). Investigation of sites of pseudorabies virus latency, using polymerase chain reaction. Am. J. Vet. Res..

[35] Tham K.M., Motha M.X., Horner G.W., Ralston J.C. (1994). Polymerase chain reaction amplification of latent aujeszky&apos;s disease virus in dexamethasone treated pigs. Arch. Virol..

[36] Capua I., Fico R., Banks M., Tamba M., Calzetta G. (1997). Isolation and characterisation of an aujeszky&apos;s disease virus naturally infecting a wild boar (sus scrofa). Vet. Microbiol..

[37] Brittle E.E., Reynolds A.E., Enquist L.W. (2004). Two modes of pseudorabies virus neuroinvasion and lethality in mice. J. Virol..

[38] Mettenleiter T.C. (2003). Pathogenesis of neurotropic herpesviruses: Role of viral glycoproteins in neuroinvasion and transneuronal spread. Virus Res..

[39] Esiri M.M. (1982). Herpes simplex encephalitis. An immunohistological study of the distribution of viral antigen within the brain. J. Neurol. Sci..

[40] Casrouge A., Zhang S.-Y., Eidenschenk C., Jouanguy E., Puel A., Yang K., Alcais A., Picard C., Mahfoufi N., Nicolas N. (2006). Herpes simplex virus encephalitis in human unc-93b deficiency. Science.

[41] Tabeta K., Hoebe K., Janssen E.M., Du X., Georgel P., Crozat K., Mudd S., Mann N., Sovath S., Goode J. (2006). The unc93b1 mutation 3d disrupts exogenous antigen presentation and signaling via toll-like receptors 3, 7 and 9. Nat. Immunol..

[42] Kim Y.-M., Brinkmann M.M., Paquet M.-E., Ploegh H.L. (2008). Unc93b1 delivers nucleotide-sensing toll-like receptors to endolysosomes. Nature.

[43] Lafaille F.G., Pessach I.M., Zhang S.Y., Ciancanelli M.J., Herman M., Abhyankar A., Ying S.W., Keros S., Goldstein P.A., Mostoslavsky G. (2012). Impaired intrinsic immunity to hsv-1 in human ipsc-derived tlr3-deficient cns cells. Nature.

[44] Conrady C.D., Drevets D.A., Carr D.J.J. (2010). Herpes simplex type i (hsv-1) infection of the nervous system: Is an immune response a good thing?. J. Neuroimmunol..

[45] Pérez de Diego R., Sancho-Shimizu V., Lorenzo L., Puel A., Plancoulaine S., Picard C., Herman M., Cardon A., Durandy A., Bustamante J. (2010). Human traf3 adaptor molecule deficiency leads to impaired toll-like receptor 3 response and susceptibility to herpes simplex encephalitis. Immunity.

[46] Zhou Y., Ye L., Wan Q., Zhou L., Wang X., Li J., Hu S., Zhou D., Ho W. (2009). Activation of toll-like receptors inhibits herpes simplex virus-1 infection of human neuronal cells. J. Neurosci. Res..

[47] Reinert L.S., Harder L., Holm C.K., Iversen M.B., Horan K.A., Dagnæs-Hansen F., Ulhøi B.P., Holm T.H., Mogensen T.H., Owens T. (2012). Tlr3 deficiency renders astrocytes permissive to herpes simplex virus infection and facilitates establishment of cns infection in mice. J. Clin. Invest..

[48] Mcgavern D.B., Kang S.S. (2011). Illuminating viral infections in the nervous system. Nat. Rev. Immunol..

[49] Mettenleiter T.C., Klupp B.G., Granzow H. (2009). Herpesvirus assembly: An update. Virus Res..

[50] WuDunn D., Spear P.G. (1989). Initial interaction of herpes simplex virus with cells is binding to heparan sulfate. J. Virol..

[51] Mettenleiter T., Zsak L., Zuckermann F., Sugg N., Kern H., Ben-Porat T. (1990). Interaction of glycoprotein giii with a cellular heparinlike substance mediates adsorption of pseudorabies virus. J. Virol..

[52] Herold B.C., Visalli R.J., Susmarski N., Brandt C.R., Spear P.G. (1994). Glycoprotein c-independent binding of herpes simplex virus to cells requires cell surface heparan sulphate and glycoprotein b. J. Gen. Virol..

[53] Laquerre S., Argnani R., Anderson D.B., Zucchini S., Manservigi R., Glorioso J.C. (1998). Heparan sulfate proteoglycan binding by herpes simplex virus type 1 glycoproteins b and c, which differ in their contributions to virus attachment, penetration, and cell-to-cell spread. J. Virol..

[54] Geraghty R.J., Krummenacher C., Cohen G.H., Eisenberg R.J., Spear P.G. (1998). Entry of alphaherpesviruses mediated by poliovirus receptor-related protein 1 and poliovirus receptor. Science.

[55] Montgomery R.I., Warner M.S., Lum B.J., Spear P.G. (1996). Herpes simplex virus-1 entry into cells mediated by a novel member of the tnf/ngf receptor family. Cell.

[56] Shukla D., Liu J., Blaiklock P., Shworak N.W., Bai X., Esko J.D., Cohen G.H., Eisenberg R.J., Rosenberg R.D., Spear P.G. (1999). A novel role for 3-o-sulfated heparan sulfate in herpes simplex virus 1 entry. Cell.

[57] Kopp S.J., Banisadr G., Glajch K., Maurer U.E., Grünewald K., Miller R.J., Osten P., Spear P.G. (2009). Infection of neurons and encephalitis after intracranial inoculation of herpes simplex virus requires the entry receptor nectin-1. Proc. Natl. Acad. Sci. USA.

[58] Peeters B., Pol J., Gielkens A., Moormann R. (1993). Envelope glycoprotein gp50 of pseudorabies virus is essential for virus entry but is not required for viral spread in mice. J. Virol..

[59] Peeters B., de Wind N., Hooisma M., Wagenaar F., Gielkens A., Moormann R. (1992). Pseudorabies virus envelope glycoproteins gp50 and gii are essential for virus penetration, but only gii is involved in membrane fusion. J. Virol..

[60] Ch'ng T.H., Spear P.G., Struyf F., Enquist L.W. (2007). Glycoprotein d-independent spread of pseudorabies virus infection in cultured peripheral nervous system neurons in a compartmented system. J. Virol..

[61] Connolly S.A., Whitbeck J.J., Rux A.H., Krummenacher C., van Drunen Littel-van den Hurk S., Cohen G.H., Eisenberg R.J. (2001). Glycoprotein d homologs in herpes simplex virus type 1, pseudorabies virus, and bovine herpes virus type 1 bind directly to human hvec(nectin-1) with different affinities. Virology.

[62] Geraghty R.J., Jogger C.R., Spear P.G. (2000). Cellular expression of alphaherpesvirus gd interferes with entry of homologous and heterologous alphaherpesviruses by blocking access to a shared gd receptor. Virology.

[63] Suenaga T., Satoh T., Somboonthum P., Kawaguchi Y., Mori Y., Arase H. (2010). Myelin-associated glycoprotein mediates membrane fusion and entry of neurotropic herpesviruses. Proc. Natl. Acad. Sci. USA.

[64] Berarducci B., Rajamani J., Reichelt M., Sommer M., Zerboni L., Arvin A.M. (2009). Deletion of the first cysteine-rich region of the varicella-zoster virus glycoprotein e ectodomain abolishes the ge and gi interaction and differentially affects cell-cell spread and viral entry. J. Virol..

[65] Li Q., Ali M.A., Cohen J.I. (2006). Insulin degrading enzyme is a cellular receptor mediating varicella-zoster virus infection and cell-to-cell spread. Cell.

[66] Li Q., Krogmann T., Ali M.A., Tang W.-J., Cohen J.I. (2007). The amino terminus of varicella-zoster virus (vzv) glycoprotein e is required for binding to insulin-degrading enzyme, a vzv receptor. J. Virol..

[67] Spear P.G., Longnecker R. (2003). Herpesvirus entry: An update. J. Virol..

[68] Chowdary T.K., Cairns T.M., Atanasiu D., Cohen G.H., Eisenberg R.J., Heldwein E.E. (2010). Crystal structure of the conserved herpesvirus fusion regulator complex gh-gl. Nat. Struct. Mol. Biol..

[69] Heldwein E.E., Krummenacher C. (2008). Entry of herpesviruses into mammalian cells. Cell Mol. Life Sci..

[70] Heldwein E.E., Lou H., Bender F.C., Cohen G.H., Eisenberg R.J., Harrison S.C. (2006). Crystal structure of glycoprotein b from herpes simplex virus 1. Science.

[71] Granzow H., Klupp B.G., Mettenleiter T. (2005). Entry of pseudorabies virus: An immunogold-labeling study. J. Virol..

[72] Luxton G.W.G., Haverlock S., Coller K.E., Antinone S.E., Pincetic A., Smith G. (2005). Targeting of herpesvirus capsid transport in axons is coupled to association with specific sets of tegument proteins. Proc. Natl. Acad. Sci. USA.

[73] Radtke K., Kieneke D., Wolfstein A., Michael K., Steffen W., Scholz T., Karger A., Sodeik B. (2010). Plus- and minus-end directed microtubule motors bind simultaneously to herpes simplex virus capsids using different inner tegument structures. PLoS Pathogens.

[74] Copeland A.M., Newcomb W.W., Brown J.C. (2009). Herpes simplex virus replication: Roles of viral proteins and nucleoporins in capsid-nucleus attachment. J. Virol..

[75] Coller K.E., Smith G. (2008). Two viral kinases are required for sustained long distance axon transport of a neuroinvasive herpesvirus. Traffic.

[76] Luxton G.W.G., Lee J.I.-H., Haverlock-Moyns S., Schober J.M., Smith G. (2006). The pseudorabies virus vp1/2 tegument protein is required for intracellular capsid transport. J. Virol..

[77] Jovasevic V., Liang L., Roizman B. (2008). Proteolytic cleavage of vp1–2 is required for release of herpes simplex virus 1 DNA into the nucleus. J. Virol..

[78] Ihara S., Feldman L., Watanabe S., Ben-Porat T. (1983). Characterization of the immediate-early functions of pseudorabies virus. Virology.

[79] Kwong A.D., Frenkel N. (1989). The herpes simplex virus virion host shutoff function. J. Virol..

[80] Ladin B.F., Blankenship M.L., Ben-Porat T. (1980). Replication of herpesvirus DNA. V. Maturation of concatemeric DNA of pseudorabies virus to genome length is related to capsid formation. J. Virol..

[81] Mettenleiter T.C. (2002). Herpesvirus assembly and egress. J. Virol..

[82] Granzow H., Weiland F., Jöns A., Klupp B.G., Karger A., Mettenleiter T. (1997). Ultrastructural analysis of the replication cycle of pseudorabies virus in cell culture: A reassessment. J. Virol..

[83] Lycke E., Hamark B., Johansson M., Krotochwil A., Lycke J., Svennerholm B. (1988). Herpes simplex virus infection of the human sensory neuron. An electron microscopy study. Arch. Virol..

[84] Speese S.D., Ashley J., Jokhi V., Nunnari J., Barria R., Li Y., Ataman B., Koon A., Chang Y.-T., Li Q. (2012). Nuclear envelope budding enables large ribonucleoprotein particle export during synaptic wnt signaling. Cell.

[85] Chang Y.E., Van Sant C., Krug P.W., Sears A.E., Roizman B. (1997). The null mutant of the u(l)31 gene of herpes simplex virus 1: Construction and phenotype in infected cells. J. Virol..

[86] Reynolds A.E., Wills E.G., Roller R.J., Ryckman B.J., Baines J.D. (2002). Ultrastructural localization of the herpes simplex virus type 1 ul31, ul34, and us3 proteins suggests specific roles in primary envelopment and egress of nucleocapsids. J. Virol..

[87] Reynolds A.E., Ryckman B.J., Baines J.D., Zhou Y., Liang L., Roller R.J. (2001). U(l)31 and u(l)34 proteins of herpes simplex virus type 1 form a complex that accumulates at the nuclear rim and is required for envelopment of nucleocapsids. J. Virol..

[88] Roller R.J., Zhou Y., Schnetzer R., Ferguson J., DeSalvo D. (2000). Herpes simplex virus type 1 u(l)34 gene product is required for viral envelopment. J. Virol..

[89] Chang Y.E., Roizman B. (1993). The product of the ul31 gene of herpes simplex virus 1 is a nuclear phosphoprotein which partitions with the nuclear matrix. J. Virol..

[90] Mou F., Forest T., Baines J.D. (2007). Us3 of herpes simplex virus type 1 encodes a promiscuous protein kinase that phosphorylates and alters localization of lamin a/c in infected cells. J. Virol..

[91] Naldinho-Souto R., Browne H., Minson T. (2006). Herpes simplex virus tegument protein vp16 is a component of primary enveloped virions. J. Virol..

[92] Padula M.E., Sydnor M.L., Wilson D.W. (2009). Isolation and preliminary characterization of herpes simplex virus 1 primary enveloped virions from the perinuclear space. J. Virol..

[93] Baines J.D., Jacob R.J., Simmerman L., Roizman B. (1995). The herpes simplex virus 1 ul11 proteins are associated with cytoplasmic and nuclear membranes and with nuclear bodies of infected cells. J. Virol..

[94] Read G.S., Patterson M. (2007). Packaging of the virion host shutoff (vhs) protein of herpes simplex virus: Two forms of the vhs polypeptide are associated with intranuclear b and c capsids, but only one is associated with enveloped virions. J. Virol..

[95] McMillan T.N., Johnson D.C. (2001). Cytoplasmic domain of herpes simplex virus ge causes accumulation in the trans-golgi network, a site of virus envelopment and sorting of virions to cell junctions. J. Virol..

[96] Harley C.A., Dasgupta A., Wilson D.W. (2001). Characterization of herpes simplex virus-containing organelles by subcellular fractionation: Role for organelle acidification in assembly of infectious particles. J. Virol..

[97] Wisner T.W., Johnson D.C. (2004). Redistribution of cellular and herpes simplex virus proteins from the trans-golgi network to cell junctions without enveloped capsids. J. Virol..

[98] Turcotte S., Letellier J., Lippé R. (2005). Herpes simplex virus type 1 capsids transit by the trans-golgi network, where viral glycoproteins accumulate independently of capsid egress. J. Virol..

[99] Campadelli G., Brandimarti R., Di Lazzaro C., Ward P.L., Roizman B., Torrisi M.R. (1993). Fragmentation and dispersal of golgi proteins and redistribution of glycoproteins and glycolipids processed through the golgi apparatus after infection with herpes simplex virus 1. Proc. Natl. Acad. Sci. USA.

[100] Kratchmarov R., Taylor M.P., Enquist L.W. (2012). Making the case: Married *versus* separate models of alphaherpes virus anterograde transport in axons. Rev. Med. Virol..

[101] Johnson D.C., Baines J.D. (2011). Herpesviruses remodel host membranes for virus egress. Nat. Rev. Microbiol..

[102] Taylor M.P., Kramer T., Lyman M.G., Kratchmarov R., Enquist L.W. (2012). Visualization of an alphaherpesvirus membrane protein that is essential for anterograde axonal spread of infection in neurons. MBio.

[103] Wisner T.W., Sugimoto K., Howard P.W., Kawaguchi Y., Johnson D.C. (2011). Anterograde transport of herpes simplex virus capsids in neurons by both separate and married mechanisms. J. Virol..

[104] Huang J., Lazear H.M., Friedman H.M. (2011). Completely assembled virus particles detected by transmission electron microscopy in proximal and mid-axons of neurons infected with herpes simplex virus type 1, herpes simplex virus type 2 and pseudorabies virus. Virology.

[105] Antinone S.E., Zaichick S.V., Smith G. (2010). Resolving the assembly state of herpes simplex virus during axon transport by live-cell imaging. J. Virol..

[106] Negatsch A., Granzow H., Maresch C., Klupp B.G., Fuchs W., Teifke J.P., Mettenleiter T.C. (2010). Ultrastructural analysis of virion formation and intraaxonal transport of herpes simplex virus type 1 in primary rat neurons. J. Virol..

[107] Maresch C., Granzow H., Negatsch A., Klupp B.G., Fuchs W., Teifke J.P., Mettenleiter T.C. (2010). Ultrastructural analysis of virion formation and anterograde intraaxonal transport of the alphaherpesvirus pseudorabies virus in primary neurons. J. Virol..

[108] Lyman M., Feierbach B., Curanovic D., Bisher M., Enquist L.W. (2007). Pseudorabies virus us9 directs axonal sorting of viral capsids. J. Virol..

[109] Feierbach B., Bisher M., Goodhouse J., Enquist L.W. (2007). *In vitro* analysis of transneuronal spread of an alphaherpesvirus infection in peripheral nervous system neurons. J. Virol..

[110] Antinone S.E., Smith G. (2006). Two modes of herpesvirus trafficking in neurons: Membrane acquisition directs motion. J. Virol..

[111] Ch'ng T.H., Enquist L.W. (2005). Efficient axonal localization of alphaherpesvirus structural proteins in cultured sympathetic neurons requires viral glycoprotein e. J. Virol..

[112] del Rio T., Ch'ng T.H., Flood E.A., Gross S.P., Enquist L.W. (2005). Heterogeneity of a fluorescent tegument component in single pseudorabies virus virions and enveloped axonal assemblies. J. Virol..

[113] Ohara P.T., Chin M.S., LaVail J.H. (2000). The spread of herpes simplex virus type 1 from trigeminal neurons to the murine cornea: An immunoelectron microscopy study. J. Virol..

[114] LaVail J.H., Topp K.S., Giblin P.A., Garner J.A. (1997). Factors that contribute to the transneuronal spread of herpes simplex virus. J. Neurosci. Res..

[115] Kristensson K., Sheppard R.D., Bornstein M.B. (1974). Observations on uptake of herpes simplex virus in organized cultures of mammalian nervous tissue. Acta. Neuropathol..

[116] Cook M.L., Stevens J.G. (1973). Pathogenesis of herpetic neuritis and ganglionitis in mice: Evidence for intra-axonal transport of infection. Infect. Immun..

[117] Yamamoto T., Otani S., Shiraki H. (1973). Ultrastructure of herpes simplex virus infection of the nervous system of mice. Acta. Neuropathol..

[118] Hill T.J., Field H.J., Roome A.P. (1972). Intra-axonal location of herpes simplex virus particles. J. Gen. Virol..

[119] Ibiricu I., Huiskonen J.T., Döhner K., Bradke F., Sodeik B., Grünewald K. (2011). Cryo electron tomography of herpes simplex virus during axonal transport and secondary envelopment in primary neurons. PLoS Pathog..

[120] Miranda-Saksena M., Boadle R.A., Aggarwal A., Tijono B., Rixon F.J., Diefenbach R.J., Cunningham A.L. (2009). Herpes simplex virus utilizes the large secretory vesicle pathway for anterograde transport of tegument and envelope proteins and for viral exocytosis from growth cones of human fetal axons. J. Virol..

[121] Snyder A., Polcicova K., Johnson D.C. (2008). Herpes simplex virus ge/gi and us9 proteins promote transport of both capsids and virion glycoproteins in neuronal axons. J. Virol..

[122] Saksena M.M., Wakisaka H., Tijono B., Boadle R.A., Rixon F., Takahashi H., Cunningham A.L. (2006). Herpes simplex virus type 1 accumulation, envelopment, and exit in growth cones and varicosities in mid-distal regions of axons. J. Virol..

[123] Snyder A., Wisner T.W., Johnson D.C. (2006). Herpes simplex virus capsids are transported in neuronal axons without an envelope containing the viral glycoproteins. J. Virol..

[124] Tomishima M.J., Enquist L.W. (2001). A conserved alpha-herpesvirus protein necessary for axonal localization of viral membrane proteins. J. Cell Biol..

[125] Miranda-Saksena M., Armati P., Boadle R.A., Holland D.J., Cunningham A.L. (2000). Anterograde transport of herpes simplex virus type 1 in cultured, dissociated human and rat dorsal root ganglion neurons. J. Virol..

[126] Holland D.J., Miranda-Saksena M., Boadle R.A., Armati P., Cunningham A.L. (1999). Anterograde transport of herpes simplex virus proteins in axons of peripheral human fetal neurons: An immunoelectron microscopy study. J. Virol..

[127] Penfold M.E., Armati P.J., Cunningham A.L. (1994). Axonal transport of herpes simplex virions to epidermal cells: Evidence for a specialized mode of virus transport and assembly. Proc. Natl. Acad. Sci. USA.

[128] Curanovic D., Enquist L.W. (2009). Directional transneuronal spread of alpha-herpesvirus infection. Future Virol..

[129] Curanovic D., Enquist L.W. (2009). Virion-incorporated glycoprotein b mediates transneuronal spread of pseudorabies virus. J. Virol..

[130] Cai H., Reinisch K., Ferro-Novick S. (2007). Coats, tethers, rabs, and snares work together to mediate the intracellular destination of a transport vesicle. Dev. Cell..

[131] Kramer T., Greco T.M., Taylor M.P., Ambrosini A.E., Cristea I.M., Enquist L.W. (2012). Kinesin-3 mediates axonal sorting and directional transport of alphaherpesvirus particles in neurons. Cell Host Microbe..

[132] Sodeik B. (2000). Mechanisms of viral transport in the cytoplasm. Trends Microbiol..

[133] Lyman M.G., Enquist L.W. (2009). Herpesvirus interactions with the host cytoskeleton. J. Virol..

[134] Favoreel H.W., Enquist L.W., Feierbach B. (2007). Actin and rho gtpases in herpesvirus biology. Trends Microbiol..

[135] Atkinson S.J., Doberstein S.K., Pollard T.D. (1992). Moving off the beaten track. Curr. Biol..

[136] Kelleher J.F., Titus M.A. (1998). Intracellular motility: How can we all work together?. Curr. Biol..

[137] Langford G.M. (1995). Actin- and microtubule-dependent organelle motors: Interrelationships between the two motility systems. Curr. Opin. Cell. Biol..

[138] Pollard T.D., Blanchoin L., Mullins R.D. (2001). Actin dynamics. J. Cell. Sci..

[139] Pfaendtner J., Lyman E., Pollard T.D., Voth G.A. (2010). Structure and dynamics of the actin filament. J. Mol. Biol..

[140] Pollard T.D., Cooper J.A. (2009). Actin, a central player in cell shape and movement. Science.

[141] Krendel M., Mooseker M.S. (2005). Myosins: Tails (and heads) of functional diversity. Physiology.

[142] Lewis T.L., Mao T., Arnold D.B. (2011). A role for myosin vi in the localization of axonal proteins. PLoS Biology.

[143] Hirokawa N., Niwa S., Tanaka Y. (2010). Molecular motors in neurons: Transport mechanisms and roles in brain function, development, and disease. Neuron..

[144] Desai A., Mitchison T.J. (1997). Microtubule polymerization dynamics. Annu. Rev. Cell. Dev. Biol..

[145] Lüders J., Stearns T. (2007). Microtubule-organizing centres: A re-evaluation. Nat. Rev. Mol. Cell. Biol..

[146] Kirschner M.W., Mitchison T. (1986). Microtubule dynamics. Nature.

[147] Mitchison T., Kirschner M. (1984). Dynamic instability of microtubule growth. Nature.

[148] Conde C., Cáceres A. (2009). Microtubule assembly, organization and dynamics in axons and dendrites. Nat. Rev. Neurosci..

[149] Vale R.D. (2003). The molecular motor toolbox for intracellular transport. Cell.

[150] Lawrence C.J., Dawe R.K., Christie K.R., Cleveland D.W., Dawson S.C., Endow S.A., Goldstein L.S.B., Goodson H.V., Hirokawa N., Howard J. (2004). A standardized kinesin nomenclature. J. Cell. Biol..

[151] Petrásek J., Schwarzerová K. (2009). Actin and microtubule cytoskeleton interactions. Curr. Opin. Plant. Biol..

[152] Arnold D.B. (2009). Actin and microtubule-based cytoskeletal cues direct polarized targeting of proteins in neurons. Sci. Signal..

[153] Song A.H., Wang D., Chen G., Li Y., Luo J., Duan S., Poo M.M. (2009). A selective filter for cytoplasmic transport at the axon initial segment. Cell.

[154] Leterrier C., Vacher H., Fache M.P., d'Ortoli S.A., Castets F., Autillo-Touati A., Dargent B. (2011). End-binding proteins eb3 and eb1 link microtubules to ankyrin g in the axon initial segment. Proc. Natl. Acad. Sci. USA.

[155] Nakata T., Hirokawa N. (2003). Microtubules provide directional cues for polarized axonal transport through interaction with kinesin motor head. J. Cell. Biol..

[156] Hedstrom K.L., Ogawa Y., Rasband M.N. (2008). Ankyring is required for maintenance of the axon initial segment and neuronal polarity. J. Cell. Biol..

[157] Sodeik B., Ebersold M.W., Helenius A. (1997). Microtubule-mediated transport of incoming herpes simplex virus 1 capsids to the nucleus. J. Cell. Biol..

[158] Clement C., Tiwari V., Scanlan P.M., Valyi-Nagy T., Yue B.Y., Shukla D. (2006). A novel role for phagocytosis-like uptake in herpes simplex virus entry. J. Cell. Biol..

[159] De Regge N., Nauwynck H.J., Geenen K., Krummenacher C., Cohen G.H., Eisenberg R.J., Mettenleiter T., Favoreel H.W. (2006). Alpha-herpesvirus glycoprotein d interaction with sensory neurons triggers formation of varicosities that serve as virus exit sites. J. Cell. Biol..

[160] Kristensson K., Lycke E., Röyttä M., Svennerholm B., Vahlne A. (1986). Neuritic transport of herpes simplex virus in rat sensory neurons *in vitro*. Effects of substances interacting with microtubular function and axonal flow [nocodazole, taxol and erythro-9–3-(2-hydroxynonyl)adenine]. J. Gen. Virol..

[161] Liu W.W., Goodhouse J., Jeon N.L., Enquist L.W. (2008). A microfluidic chamber for analysis of neuron-to-cell spread and axonal transport of an alpha-herpesvirus. PLoS ONE.

[162] Smith G., Pomeranz L., Gross S.P., Enquist L.W. (2004). Local modulation of plus-end transport targets herpesvirus entry and egress in sensory axons. Proc. Natl. Acad. Sci. USA.

[163] Kardon J.R., Vale R.D. (2009). Regulators of the cytoplasmic dynein motor. Nat. Rev. Mol. Cell. Biol..

[164] Douglas M.W., Diefenbach R.J., Homa F.L., Miranda-Saksena M., Rixon F.J., Vittone V., Byth K., Cunningham A.L. (2004). Herpes simplex virus type 1 capsid protein vp26 interacts with dynein light chains rp3 and tctex1 and plays a role in retrograde cellular transport. J. Biol. Chem..

[165] Roberts A.P.E., Abaitua F., O'Hare P., McNab D., Rixon F.J., Pasdeloup D. (2009). Differing roles of inner tegument proteins pul36 and pul37 during entry of herpes simplex virus type 1. J. Virol..

[166] Schipke J., Pohlmann A., Diestel R., Binz A., Rudolph K., Nagel C.-H., Bauerfeind R., Sodeik B. (2012). The c terminus of the large tegument protein pul36 contains multiple capsid binding sites that function differently during assembly and cell entry of herpes simplex virus. J. Virol..

[167] Shanda S.K., Wilson D.W. (2008). Ul36p is required for efficient transport of membrane-associated herpes simplex virus type 1 along microtubules. J. Virol..

[168] Abaitua F., Daikoku T., Crump C., Bolstad M., O'Hare P. (2011). A single mutation responsible for temperature-sensitive entry and assembly defects in the vp1–2 protein of herpes simplex virus. J. Virol..

[169] Abaitua F., Souto R.N., Browne H., Daikoku T., O'Hare P. (2009). Characterization of the herpes simplex virus (hsv)-1 tegument protein vp1–2 during infection with the hsv temperature-sensitive mutant tsb7. J. Gen. Virol..

[170] Antinone S.E., Shubeita G.T., Coller K.E., Lee J.I., Haverlock-Moyns S., Gross S.P., Smith G. (2006). The herpesvirus capsid surface protein, vp26, and the majority of the tegument proteins are dispensable for capsid transport toward the nucleus. J. Virol..

[171] Desai P., DeLuca N.A., Person S. (1998). Herpes simplex virus type 1 vp26 is not essential for replication in cell culture but influences production of infectious virus in the nervous system of infected mice. Virology.

[172] Döhner K., Radtke K., Schmidt S., Sodeik B. (2006). Eclipse phase of herpes simplex virus type 1 infection: Efficient dynein-mediated capsid transport without the small capsid protein vp26. J. Virol..

[173] Desai P.J. (2000). A null mutation in the ul36 gene of herpes simplex virus type 1 results in accumulation of unenveloped DNA-filled capsids in the cytoplasm of infected cells. J. Virol..

[174] Fuchs W., Klupp B.G., Granzow H., Mettenleiter T. (2004). Essential function of the pseudorabies virus ul36 gene product is independent of its interaction with the ul37 protein. J. Virol..

[175] Vittone V., Diefenbach E., Triffett D., Douglas M.W., Cunningham A.L., Diefenbach R.J. (2005). Determination of interactions between tegument proteins of herpes simplex virus type 1. J. Virol..

[176] Lee J.H., Vittone V., Diefenbach E., Cunningham A.L., Diefenbach R.J. (2008). Identification of structural protein-protein interactions of herpes simplex virus type 1. Virology.

[177] Loomis J.S., Bowzard J.B., Courtney R.J., Wills J.W. (2001). Intracellular trafficking of the ul11 tegument protein of herpes simplex virus type 1. J. Virol..

[178] Wagenaar F., Pol J.M., Peeters B., Gielkens A.L., de Wind N., Kimman T.G. (1995). The us3-encoded protein kinase from pseudorabies virus affects egress of virions from the nucleus. J. Gen. Virol..

[179] Klupp B.G., Böttcher S., Granzow H., Kopp M., Mettenleiter T. (2005). Complex formation between the ul16 and ul21 tegument proteins of pseudorabies virus. J. Virol..

[180] Kramer T., Greco T.M., Enquist L.W., Cristea I.M. (2011). Proteomic characterization of pseudorabies virus extracellular virions. J. Virol..

[181] Coller K.E., Lee J.I.-H., Ueda A., Smith G. (2007). The capsid and tegument of the alphaherpesviruses are linked by an interaction between the ul25 and vp1/2 proteins. J. Virol..

[182] Diefenbach R.J., Miranda-Saksena M., Diefenbach E., Holland D.J., Boadle R.A., Armati P.J., Cunningham A.L. (2002). Herpes simplex virus tegument protein us11 interacts with conventional kinesin heavy chain. J. Virol..

[183] Benboudjema L., Mulvey M., Gao Y., Pimplikar S.W., Mohr I. (2003). Association of the herpes simplex virus type 1 us11 gene product with the cellular kinesin light-chain-related protein pat1 results in the redistribution of both polypeptides. J. Virol..

[184] Lee G.E., Murray J.W., Wolkoff A.W., Wilson D.W. (2006). Reconstitution of herpes simplex virus microtubule-dependent trafficking *in vitro*. J. Virol..

[185] Fuchs W., Granzow H., Klupp B.G., Kopp M., Mettenleiter T. (2002). The ul48 tegument protein of pseudorabies virus is critical for intracytoplasmic assembly of infectious virions. J. Virol..

[186] Wolfstein A., Nagel C.-H., Radtke K., Döhner K., Allan V.J., Sodeik B. (2006). The inner tegument promotes herpes simplex virus capsid motility along microtubules *in vitro*. Traffic.

[187] Desai P., Sexton G.L., Huang E., Person S. (2008). Localization of herpes simplex virus type 1 ul37 in the golgi complex requires ul36 but not capsid structures. J. Virol..

[188] Szilágyi J.F., Cunningham C. (1991). Identification and characterization of a novel non-infectious herpes simplex virus-related particle. J. Gen. Virol..

[189] Babic N., Klupp B., Brack A., Mettenleiter T., Ugolini G., Flamand A. (1996). Deletion of glycoprotein ge reduces the propagation of pseudorabies virus in the nervous system of mice after intranasal inoculation. Virology.

[190] Brideau A.D., Card J.P., Enquist L.W. (2000). Role of pseudorabies virus us9, a type ii membrane protein, in infection of tissue culture cells and the rat nervous system. J. Virol..

[191] Card J.P., Levitt P., Enquist L.W. (1998). Different patterns of neuronal infection after intracerebral injection of two strains of pseudorabies virus. J. Virol..

[192] Kritas S.K., Nauwynck H.J., Pensaert M.B. (1995). Dissemination of wild-type and gc-, ge-and gi-deleted mutants of aujeszky&apos;s disease virus in the maxillary nerve and trigeminal ganglion of pigs after intranasal inoculation. J. Gen. Virol..

[193] Mulder W., Pol J., Kimman T., Kok G., Priem J., Peeters B. (1996). Glycoprotein d-negative pseudorabies virus can spread transneuronally via direct neuron-to-neuron transmission in its natural host, the pig, but not after additional inactivation of ge or gi. J. Virol..

[194] Mulder W.A., Jacobs L., Priem J., Kok G.L., Wagenaar F., Kimman T.G., Pol J.M. (1994). Glycoprotein ge-negative pseudorabies virus has a reduced capability to infect second- and third-order neurons of the olfactory and trigeminal routes in the porcine central nervous system. J. Gen. Virol..

[195] Whealy M.E., Card J.P., Robbins A.K., Dubin J.R., Rziha H.J., Enquist L.W. (1993). Specific pseudorabies virus infection of the rat visual system requires both gi and gp63 glycoproteins. J. Virol..

[196] Kritas S.K., Pensaert M.B., Mettenleiter T. (1994). Role of envelope glycoproteins gi, gp63 and giii in the invasion and spread of aujeszky's disease virus in the olfactory nervous pathway of the pig. J. Gen. Virol..

[197] Ch'ng T.H., Enquist L.W. (2005). Neuron-to-cell spread of pseudorabies virus in a compartmented neuronal culture system. J. Virol..

[198] Tirabassi R.S., Enquist L.W. (1998). Role of envelope protein ge endocytosis in the pseudorabies virus life cycle. J. Virol..

[199] Tirabassi R.S., Townley R.A., Eldridge M.G., Enquist L.W. (1997). Characterization of pseudorabies virus mutants expressing carboxy-terminal truncations of ge: Evidence for envelope incorporation, virulence, and neurotropism domains. J. Virol..

[200] Brideau A.D., Enquist L., Tirabassi R.S. (2000). The role of virion membrane protein endocytosis in the herpesvirus life cycle. J. Clin. Virol..

[201] Tirabassi R.S., Enquist L.W. (1999). Mutation of the yxxl endocytosis motif in the cytoplasmic tail of pseudorabies virus ge. J. Virol..

[202] Brideau A.D., Banfield B.W., Enquist L.W. (1998). The us9 gene product of pseudorabies virus, an alphaherpesvirus, is a phosphorylated, tail-anchored type ii membrane protein. J. Virol..

[203] Lyman M.G., Curanovic D., Enquist L.W. (2008). Targeting of pseudorabies virus structural proteins to axons requires association of the viral us9 protein with lipid rafts. PLoS Pathog..

[204] Tomishima M.J., Smith G., Enquist L.W. (2001). Sorting and transport of alpha herpesviruses in axons. Traffic.

[205] Aizawa H., Sekine Y., Takemura R., Zhang Z., Nangaku M., Hirokawa N. (1992). Kinesin family in murine central nervous system. J. Cell. Biol..

[206] Okada Y., Yamazaki H., Sekine-Aizawa Y., Hirokawa N. (1995). The neuron-specific kinesin superfamily protein kif1a is a unique monomeric motor for anterograde axonal transport of synaptic vesicle precursors. Cell.

[207] Lo K.Y., Kuzmin A., Unger S.M., Petersen J.D., Silverman M.A. (2011). Kif1a is the primary anterograde motor protein required for the axonal transport of dense-core vesicles in cultured hippocampal neurons. Neurosci. Lett..

[208] Pack-Chung E., Kurshan P.T., Dickman D.K., Schwarz T.L. (2007). A drosophila kinesin required for synaptic bouton formation and synaptic vesicle transport. Nat. Neurosci..

[209] Yonekawa Y., Harada A., Okada Y., Funakoshi T., Kanai Y., Takei Y., Terada S., Noda T., Hirokawa N. (1998). Defect in synaptic vesicle precursor transport and neuronal cell death in kif1a motor protein-deficient mice. J. Cell. Biol..

[210] Hall D.H., Hedgecock E.M. (1991). Kinesin-related gene unc-104 is required for axonal transport of synaptic vesicles in c. Elegans. Cell.

[211] Klopfenstein D.R., Tomishige M., Stuurman N., Vale R.D. (2002). Role of phosphatidylinositol(4,5)bisphosphate organization in membrane transport by the unc104 kinesin motor. Cell.

[212] Smith G., Gross S.P., Enquist L.W. (2001). Herpesviruses use bidirectional fast-axonal transport to spread in sensory neurons. Proc. Natl. Acad. Sci. USA.

[213] Tannous R., Grose C. (2011). Calculation of the anterograde velocity of varicella-zoster virions in a human sciatic nerve during shingles. J. Infect. Dis..

[214] Roberts K.L., Baines J.D. (2010). Myosin va enhances secretion of herpes simplex virus 1 virions and cell surface expression of viral glycoproteins. J. Virol..

[215] Hirokawa N., Noda Y., Tanaka Y., Niwa S. (2009). Kinesin superfamily motor proteins and intracellular transport. Nat. Rev. Mol. Cell. Biol..

[216] Favoreel H.W., Van Minnebruggen G., Adriaensen D., Nauwynck H.J. (2005). Cytoskeletal rearrangements and cell extensions induced by the us3 kinase of an alphaherpesvirus are associated with enhanced spread. Proc. Natl. Acad. Sci. USA.

[217] Liu M., Schmidt E.E., Halford W.P. (2010). Icp0 dismantles microtubule networks in herpes simplex virus-infected cells. PLoS ONE.

[218] Macaskill A.F., Rinholm J.E., Twelvetrees A.E., Arancibia-Carcamo I.L., Muir J., Fransson A., Aspenstrom P., Attwell D., Kittler J.T. (2009). Miro1 is a calcium sensor for glutamate receptor-dependent localization of mitochondria at synapses. Neuron.

[219] Li Z., Okamoto K.-I., Hayashi Y., Sheng M. (2004). The importance of dendritic mitochondria in the morphogenesis and plasticity of spines and synapses. Cell.

[220] Wang X., Schwarz T.L. (2009). The mechanism of ca2+ -dependent regulation of kinesin-mediated mitochondrial motility. Cell.

[221] Grubb M.S., Burrone J. (2010). Activity-dependent relocation of the axon initial segment fine-tunes neuronal excitability. Nature.

[222] Kuba H., Oichi Y., Ohmori H. (2010). Presynaptic activity regulates na(+) channel distribution at the axon initial segment. Nature.

[223] Wimmer V.C., Reid C.A., So E.Y.-W., Berkovic S.F., Petrou S. (2010). Axon initial segment dysfunction in epilepsy. J. Physiol..

[224] Sheng Z.-H., Cai Q. (2012). Mitochondrial transport in neurons: Impact on synaptic homeostasis and neurodegeneration. Nat. Rev. Neurosci..

[225] McCarthy K.M., Tank D.W., Enquist L.W. (2009). Pseudorabies virus infection alters neuronal activity and connectivity *in vitro*. PLoS Patho..

[226] Kramer T., Enquist L.W. (2012). Alphaherpesvirus infection disrupts mitochondrial transport in neurons. Cell Host Microbe..

[227] Pivovarova N.B., Andrews S.B. (2010). Calcium-dependent mitochondrial function and dysfunction in neurons. FEBS J..

[228] Castanier C., Garcin D., Vazquez A., Arnoult D. (2010). Mitochondrial dynamics regulate the rig-i-like receptor antiviral pathway. EMBO Rep..

